# Refinement of Active and Passive Membrane Properties of Layer V Pyramidal Neurons in Rat Primary Motor Cortex During Postnatal Development

**DOI:** 10.3389/fnmol.2021.754393

**Published:** 2021-12-01

**Authors:** Patricia Perez-García, Ricardo Pardillo-Díaz, Noelia Geribaldi-Doldán, Ricardo Gómez-Oliva, Samuel Domínguez-García, Carmen Castro, Pedro Nunez-Abades, Livia Carrascal

**Affiliations:** ^1^Department of Physiology, School of Pharmacy, University of Seville, Seville, Spain; ^2^Division of Physiology, School of Medicine, University of Cádiz, Cádiz, Spain; ^3^Biomedical Research and Innovation Institute of Cádiz (INiBICA), Cádiz, Spain; ^4^Department of Human Anatomy and Embriology, University of Cádiz, Cádiz, Spain

**Keywords:** development, motor cortex, motor neurons, pyramidal neurons, membrane properties, patch clamp

## Abstract

Achieving the distinctive complex behaviors of adult mammals requires the development of a great variety of specialized neural circuits. Although the development of these circuits begins during the embryonic stage, they remain immature at birth, requiring a postnatal maturation process to achieve these complex tasks. Understanding how the neuronal membrane properties and circuits change during development is the first step to understand their transition into efficient ones. Thus, using whole cell patch clamp recordings, we have studied the changes in the electrophysiological properties of layer V pyramidal neurons of the rat primary motor cortex during postnatal development. Among all the parameters studied, only the voltage threshold was established at birth and, although some of the changes occurred mainly during the second postnatal week, other properties such as membrane potential, capacitance, duration of the post-hyperpolarization phase or the maximum firing rate were not defined until the beginning of adulthood. Those modifications lead to a decrease in neuronal excitability and to an increase in the working range in young adult neurons, allowing more sensitive and accurate responses. This maturation process, that involves an increase in neuronal size and changes in ionic conductances, seems to be influenced by the neuronal type and by the task that neurons perform as inferred from the comparison with other pyramidal and motor neuron populations.

## Introduction

The development of a large variety of specialized neuronal circuits is needed to achieve all the complex behaviors found in adult mammals. These neuronal circuits are mostly immature at birth and postnatal refinement is required in both neuronal properties and connectivity to effectively manage such a complicated task (Kriegstein et al., 1987; McCormick and Prince, 1987; Burgard and Hablitz, 1993; Ramoa and McCormick, 1994; Picken Bahrey and Moody, 2003; Carrascal et al., 2006, 2009; Smith and Brownstone, 2020). The degree to which these mature electrophysiological properties are determined by cell-intrinsic *vs*. extrinsic factors is not clear. Thus, in order to understand how neural circuits are ultimately tuned, it is necessary to investigate how the properties of their neurons develop.

The neocortex is the largest part of the mammalian brain, and it is the seat of higher cognitive functions, including fine motor tasks. Accordingly, defects in neocortical development commonly result in severe intellectual, motor and social deficits. Therefore, understanding the mechanisms that underlie the development of the neocortex facilitates the comprehension of its evolution and disease and the disclosure of their triggering mechanisms [reviewed in Borrell (2019)]. In the mammalian neocortex different cell types have been described, based on dendritic and axonal arborization patterns (Amaral and Strick, 2013). The two general cell types, pyramidal and non-pyramidal, represent projection neurons and interneurons, respectively, (Molnár and Cheung, 2006; Molyneaux et al., 2007; Amaral and Strick, 2013). Pyramidal neurons generally have long axonal projections to distant cortical and subcortical targets and are therefore thought to be important in communication between neural networks (Yuste, 2005; Chomiak et al., 2008; Chomiak and Hu, 2013), capable of supplying information that can be rapidly and synchronously reconstituted across remote sensorimotor networks (Chomiak and Hu, 2013). Neocortical neurons undergo an extensive change in their dendritic morphology during postnatal development (Petit et al., 1988; Kasper et al., 1994) and few studies in layer V pyramidal neurons from neocortex demonstrate postnatal changes in its electrophysiological properties during the first 4 postnatal weeks (Zhu, 2000; Zhang, 2004; Christophe et al., 2005; Etherington and Williams, 2011; Elston and Fujita, 2014). However, these studies are mainly focused in prefrontal and visual cortex or somatosensorial cortex. In this work we hypothesize that layer V pyramidal neurons from the primary motor cortex are tuned during postnatal development, and we wonder whether the temporal course of such changes is similar to that of other pyramidal neurons or on the contrary each population of pyramidal neurons evolves with a different temporal course dependent on their final tasks and inputs. Furthermore, previous works from our laboratory have demonstrated considerable postnatal changes in electrophysiological and morphological properties of oculomotor and genioglossal motor neurons during the first month following birth (Nunez-Abades et al., 1993; Nunez-Abades et al., 1994; Nunez-Abades and Cameron, 1995; Carrascal et al., 2006; Carrascal et al., 2009; Carrascal et al., 2010). Additionally, it has also been reported in detail the electrophysiological postnatal maturation of cervical and lumbar motor neurons showing great changes (Smith and Brownstone, 2020).

The aim of this work was to study, through whole cell patch clamp techniques in brain slices, if electrophysiological properties of layer V pyramidal neurons from the primary motor cortex are established at birth or they are refined during the first month of age, with special attention to the temporal course of maturation.

## Materials and Methods

### Animals and Brain Slices Preparation

Wistar rats of both sexes and aged between days 2 and 44 postnatal were used in the study. Rats were classified into four groups for the purpose of including data in a table: newborn (P2–P7); infantile (P11–P17); young adult (P22–P30), and adult (P31–44). A total of 23 animals were used in this study (P1–10, *N* = 5; P11–20, *N* = 5; P21–30; *N* = 7; P21–44, *N* = 6). Only one cell was recorded per slice, using 2–4 slices per animal. Experiments were performed strictly following the recommendations given by the Guide for the Care and Use of Laboratory Animals of the European Community Directive 2010/63/UE and the Spanish Royal Decree 53/2013. In addition, all the procedures were approved by the Animal Ethics Committee of the University of Seville. Overall, newborn rats were sacrificed directly by decapitation, while infantile, young adult, and adult rats were previously sure with sodium pentobarbital (50 mg/kg), ensuring that both the retraction reflex of the limb and the blinking reflex disappeared, and perfused with ice-cold low-calcium artificial cerebral spinal fluid (ACSF), to obtain the best possible preservation of the brain tissue. To guarantee that the results are not influenced by the anesthesia, two infantile animals were directly decapitated using slices obtained from these rats, 5 cells were recorded, and no significant differences were found with respect to the cells recorded from anesthetized rats of the infantile group. In all cases, the brain was rapidly extracted and placed in a dish with the same cutting solution, where the cerebellum and the most rostral part of the telencephalon was removed. The part of the brain that contained the area of interest -i.e., the motor cortex- was cut transversely into 300 μm slices using a vibratome (Leica VT1000 S, Leica Biosystems, United Kingdom). The slices were incubated in a chamber containing ACSF at 33°C for 30 min and then maintained in the same chamber at room temperature until use. The composition of the ACSF was as follows (data in mM): 126 NaCl, 2 KCl, 1.25 Na_2_HPO_4_, 26 NaHCO_3_, 10 glucose, 2 MgCl_2_, and 2 CaCl_2_; ACSF used for brain perfusion, extraction and slicing contained a lower concentration of calcium and a higher concentration of magnesium (data in mM): 4 MgCl_2_, 0.1 CaCl_2_. Both ACSF and low-calcium-ACSF solutions were bubbled with 95% O_2_ – 5% CO_2_ (pH 7.4, adjusted with HCl).

### Whole-Cell Patch Clamp Recordings and Analysis

Single slices were transferred to the recording chamber, where they were continuously superfused with aerated ACSF (∼33°C) at 1 ml/min using a peristaltic pump (Harvard Apparatus MPII, Holliston, MA, United States). As visual guidance, a Nikon Eclipse FN1 microscope equipped with infrared-differential interference contrast (IR-DIC) optics, a 40 × water immersion objective and a WAT-902H2 Ultimate Camera was used. Patch clamp micropipettes were obtained from borosilicate glass capillaries (inner diameter 0.6 mm, outer diameter 1 mm, Narishige) and a vertical puller (PC-10, Narishige, Tokyo, Japan), adjusting its resistance to 3–6 MΩ. Micropipettes were filled with a K^+^-gluconate solution (in mM): 120 K-gluconate, 10 KCl, 10 phosphocreatine disodium salt, 2 MgATP, 0.3 NaGTP, 0.1 EGTA, 10 HEPES. pH was adjusted to 7.3 using KOH and an osmolarity of 285 mosmol/kg was adjusted with sucrose. Using a micromanipulator (MP-225, Sutter Instrument, CA, United States), micropipettes were visually placed in the motor cortex area. Within this region the pyramidal neurons were identified based on their characteristic morphology (Pardillo-Díaz et al., 2016; Carrascal et al., 2020). An amplified (MultiClamp 700B, Axon Instruments, Molecular Devices, Sunnyvale, CA, United States) was also used to achieve the whole-cell patch clamp configuration. Recordings were low-pass Bessel-filtered at 10 kHz; data were digitized at 2–20 kHz using a Digidata 1550 analog-to-digital converter and acquired with the pCLAMP 10 software (Molecular Devices). For the data analysis, Clampfit 10.4 software (Molecular Devices) was used.

As performed in previous studies from our laboratory (Torres-Torrelo et al., 2012; Pardillo-Díaz et al., 2015), both passive and active properties of the cells were studied: resting membrane potential, input resistance, membrane time constant, rheobase, voltage threshold, depolarization voltage, action potential amplitude and duration, AHP duration, maximum frequency of discharge, frequency gain, and cancelation current. All cells selected for analysis had a stable resting membrane potential and did not show spontaneous action potentials.

The resting membrane potential was calculated as the difference between the intracellular and extracellular potential after the removal of the recording electrode from the cell. The input resistance was measured by injecting positive and negative square current pulses (500 ms, 1 Hz, with 10 pA increments) and calculated offline as the slope of the current-voltage relationship, following Ohm’s law. Membrane time constant was defined as the time needed for the membrane voltage to reach 2/3 of its final value. When there was evidence of inward rectification (*sag*) the voltage value achieved at the peak was used for the calculation of input resistance. For the measurement of the membrane time constant, single pulses of −30 pA were used, a current intensity that did not elicit inward rectification. Rheobase was defined as the minimum intensity of current necessary to provoke an action potential in 50% of the cases; it was calculated by applying square pulses of 100 ms, 1 Hz, with 5–20 pA increments.

The voltage threshold was the minimum voltage required by the cell to trigger an action potential (spike threshold), while the voltage depolarization was the increase in membrane potential needed to reach the voltage threshold. Thus, the depolarization voltage could be calculated as the difference between the voltage threshold and the resting membrane potential. To determine the spike threshold, the action potential recording was differentiated, with the spike onset taken as the value of the membrane potential at which the first derivative exceeded 10 V/s (Carrascal et al., 2006). Action potential amplitude was calculated as the difference between the spike voltage peak and the threshold voltage, while its duration was calculated as the width of the action potential at half its amplitude. Duration of afterhyperpolarization phase (AHP) was measured as the time elapsed between the repolarizing phase at the resting potential level and the point in which this potential is totally recovered.

Repetitive firing properties were evoked by the application of depolarizing current steps (1 s, 0.5 Hz, with 10–50 pA increments). The maximum firing frequency was taken as the highest number of spikes that cells could achieve during the repetitive discharge, independently of the current intensity. Frequency gain was defined as the slope of the relationship between firing frequency and applied current. All the neurons from this study achieved a plateau after the reaching of the maximum frequency that was not consider for the calculation of this parameter. Cancelation current was the intensity of the 1s depolarizing pulse at which the neuron began to fail in the discharge, after reaching its maximum frequency.

To confirm that soma morphology in IR-DIC was an appropriate neuronal selection method for our study, we chose a random sample of neurons within the studied population to verify their morphology through a dye-filling technique. In total, 6 neurons were intracellularly labeled with neurobiotin after performing electrophysiological recordings. For this purpose, iontophoretic injection of 1% neurobiotin (Neurobiotin tracer; Vector Laboratories, Burlingame, CA, United States) contained in the internal pipette solution was carried out by applying current steps of 400 pA of 500 ms at 0.5 Hz for 20 min. Slices containing labeled cells were deposited in a 4% paraformaldehyde solution at 4°C and left overnight, then transferred to 30% sucrose in phosphate buffer at 4°C overnight. Thereafter, dye-filled neurons were revealed with monoclonal antibiotin FITC conjugated antibody (1:120) from Sigma (Darmstadt, Germany). A Zeiss LSM 900 Airyscan 2 confocal microscope was used for the visualization and reconstruction of the labeled pyramidal cells. Stacks of 30–70 photographs were performed, using a 1 μm interval. Images were processed with ZEN 3.2 Blue Edition software. Neurons were reconstructed using the Neurolucida 360 version 2020 3.1 system (MicroBrightField, Williston, VT, United States).

### Statistical Analysis

All statistical analyses were carried out on the raw data. Results were expressed as the mean ± standard error of the mean; n indicates the number of cells included; N indicates the number of animals. GraphPad Prism software was used for the statistical calculations. A normality test (Shapiro-Wilk Test) was applied in order to check the data distribution. A variance analysis (ANOVA) with repeated measures was used to compare the means between groups. If significant differences were shown, Bonferroni test was carried out to make paired comparisons between groups. In all cases, a 95% confidence interval was considered, therefore two groups were statistically different if *p* ≤ 0.05. Correlation among two variables was measured using Pearson’s correlation coefficient (r). Fisher’s exact test was used to determine if there were non-random associations between two categorical variables (presence of sag and age groups). In figures and tables, asterisks (^∗^) indicate statistical dissimilarities between consecutive age groups, while crosses (+) indicate statistical differences when comparing first and last age groups. Finally, black lines in figures represent the best fit to the raw data.

## Results

**FIGURE 1:**
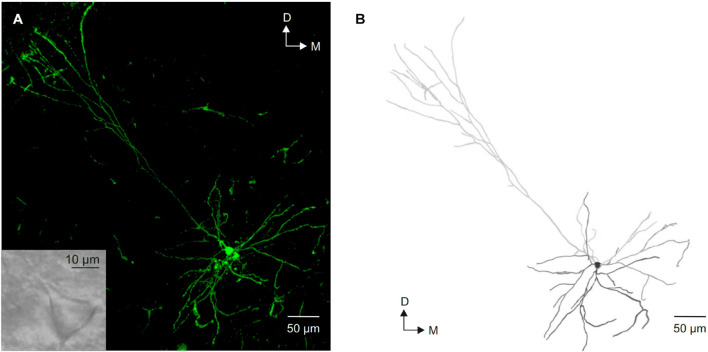
Morphological identification of layer V pyramidal neurons from the motor cortex. **(A)** Photomicrograph of a intracellular labeled layer V pyramidal neuron showing its typical morphology. The inset shows an image of this neuron under infrared microscope visualization. **(B)** Neurolucida reconstruction of the neuron shown in **(A)**. D, dorsal; M, medial.

All neurons included were identified as layer V pyramidal neurons based on their morphological characteristics (Figure 1), a large triangular-shaped cell body with a prominent apical dendrite projecting vertically to the pial surface and smaller basal dendrites. Cells were discarded if they did not present a stable resting membrane potential or if they were not silent at rest.

### Passive Membrane Properties of Layer V Pyramidal Neurons From the Motor Cortex During Postnatal Development

**FIGURE 2:**
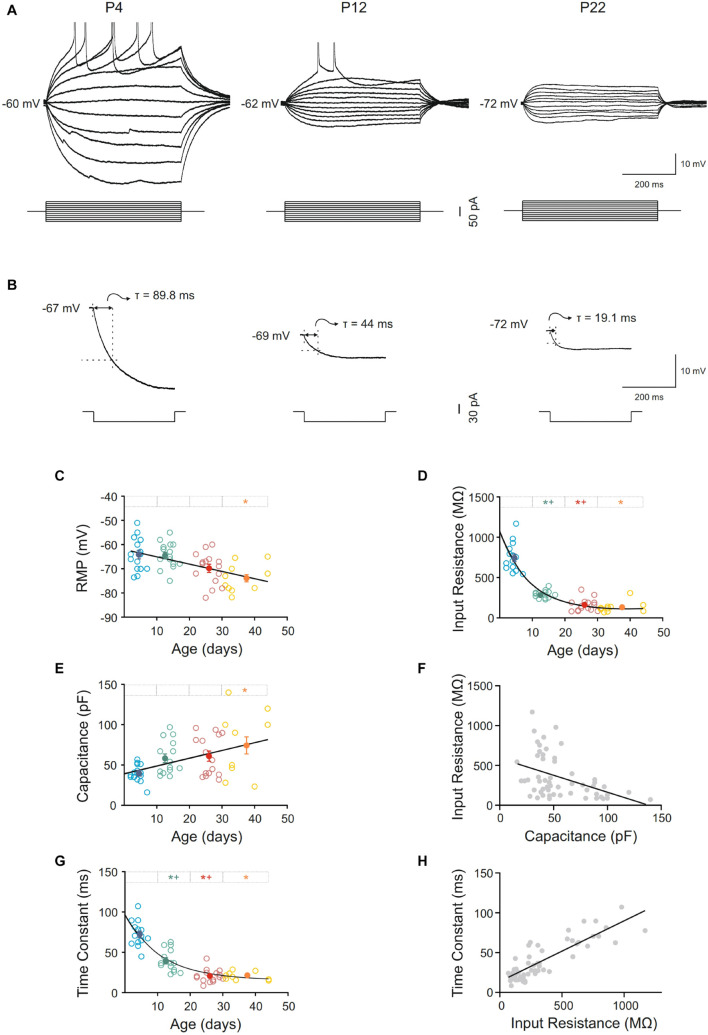
Postnatal development of passive membrane properties of motor cortex pyramidal neurons. **(A)** Membrane voltage responses to depolarizing and hyperpolarizing current pulses in representative neurons of different ages. **(B)** Average (*n* = 10) of the membrane voltage response to a hyperpolarizing current pulse (300 ms, –20 pA) in the representative neurons show in **(A)**. **(C–E,G)** Scatter plots showing the changes in resting membrane potential (RMP) **(C)**, input resistance **(D)**, capacitance **(E),** and time constant **(G)** as a function of postnatal age. Open symbols represent the raw data, while filled symbols are the means of the four different age groups and their standard errors. Each age group is represented by a color (blue = newborn, *n* = 15; green = infantile, *n* = 15; red = young adult, *n* = 15; yellow = adult, *n* = 10). An asterisk indicates a significant difference from the newborn group; a cross indicates a difference between adjacent groups. The significance level was established as *p* ≤ 0.05 **(F,H)**. Correlation between input resistance and capacitance, r = –0.43; *p* < 0.0009 **(F)** and time constant and input resistance, r = 0.86; *p* < 0.0001 **(H)**.

Pyramidal neurons from the motor cortex exhibited a stable membrane potential that progressively hyperpolarized during postnatal development. The best fit to the raw data was a linear regression (slope = −0.30 mV/day; r = 0,51; *p* < 0.0001; Figure 2C). This difference was statistically significant when the mean of newborn (−64.10 ± 1.75 mV) and adult (−73.90 ± 1.49 mV) age groups were compared (Figures 2A,C and Table 1). Input resistance also changed with postnatal maturation. The input resistance of neurons from the newborn group were the highest, being 2.6-fold greater than those of the infantile group and 5–6-fold greater than those of the young adult and adult groups (Figure 2A). All the statistical comparisons among group means showed significant differences except the two older ones, meaning that input resistance was established at the third week (Figure 2D and Table 1). Those changes are also observable in Figure 2D, which depicts the raw data (open symbols) fitting a single exponential decay (r = 0.90; τ = 8.572 days). The time constant of this fit demonstrates that the major changes of this parameter occur between the first and the beginning of the second week after birth. This fact is also supported by the comparison between the means of the newborn and the infantile groups: 744.59 ± 46.10 MΩ and 285.40 ± 12.67 MΩ, respectively (Table 1). As shown in Figure 2A, the voltage response to hyperpolarizing current pulses was nearly linear and exponentially approached a steady-state level. However, although this linearity was observable in all groups, the neurons from adult, young adult and infantile groups exhibited a membrane potential rectification at the more hyperpolarizing current pulse. This depolarizing shift in membrane potential or sag was voltage dependent and more evident at more hyperpolarizing currents (see in Figure 2A). When we examined the presence or absence of sag in the four groups, we found that only the 6,67% of neurons from the newborn group showed sag, while the 66,67% of neurons from the infantile, the 80% of neurons from the young adult, and the 85,71% neurons from the adult group exhibited sag. These differences were significant when the three older groups were compared with the newborn group without differences neither between infantile and young adult groups nor between young adult and adult groups (Table 1).

**TABLE 1:** Electrophysiological properties of pyramidal neurons from the motor cortex during postnatal development.

Membrane properties	Newborn *n* = 15	Infantile *n* = 15	Young adult *n* = 15	Adult *n* = 10
Capacitance (pF)	39.80 ± 2.67	58.40 ± 5.29	61.13 ± 6.36	74.30 ± 10.51*
Membrane Resting Potential (mV)	−64.10 ± 1.75	−64.50 ± 1.37	−69.80 ± 1.67	−73.90 ± 1.49*
Input Resistance (MΩ)	744.59 ± 46.10	285.40 ± 12.67*^+^	161.80 ± 20.09*^+^	133.40 ± 17.37*
Time Constant (ms)	72.40 ± 3.98	39.50 ± 3.40*^+^	20.80 ± 2.10*^+^	21.50 ± 1.38*
Rheobase (pA)	34.67 ± 4.94	110.00 ± 14.65*^+^	197.30 ± 26.32*^+^	209.00 ± 13.26*
Voltage Depolarization (mV)	21.67 ± 2.05	23.90 ± 1.46	33.20 ± 1.78*^+^	31.20 ± 1.33*
Voltage Threshold (mV)	−43.50 ± 2.83	−42.00 ± 1.12	−36.70 ± 2.31	−42.80 ± 1.63
Action Potential Amplitude (mV)	92.40 ± 0.96	104.10 ± 2.92*^+^	121.10 ± 2.69*^+^	118.80 ± 1.31*
Action Potential Duration (ms)	2.98 ± 0.16	1.96 ± 0.11*^+^	1.47 ± 0.11*^+^	1.48 ± 0.06*
Ahp Duration (ms)	264.68 ± 16.60	270.44 ± 23.49	159.39 ± 13.85*^+^	144.23 ± 11.61*
Frequency Gain (AP ⋅ s^−1^⋅ nA^−1^)	91.11 ± 6.47	53.81 ± 6.98*^+^	48.30 ± 4.50*	33.64 ± 3.04*
Maximum Frequency (AP ⋅ s^−1^)	18.07 ± 1.35	20.60 ± 2.25	33.93 ± 3.66*^+^	32.30 ± 2.95*
Cancelation Current (pA)	174.33 ± 17.24	437.00 ± 64.57	987.00 ± 105.50*^+^	1060.00 ± 101.62*
Cells with Sag (%)	6.67	66.67*	80.00*	85.71*

*An asterisk indicates a significant difference from the newborn group; a cross indicates a difference between adjacent groups. The significance level was established as p ≤ 0.05. All data are presented as mean ± standard error of the mean.*

*AP, action potential.*

Membrane capacitance increased almost 2-fold during maturation. This increase was progressive and only significant differences were found when the adult group was compared with the newborn one, which is consistent with a linear regression as the best fit for the raw data (slope = 0.97 pF/day; r = 0.43; *p* = 0.001; Figure 2E and Table 1). When we analyzed if membrane capacitance co-varied with input resistance, as expected by the neuronal cable theory, a weak but significant linear relationship was found (r = 0.43; *p* = 0.0009; Figure 2F). Hence, membrane capacitance increase is not the sole cause of the input resistance reduction. As membrane capacitance is a measure of neuron size, this indicates that motor cortex pyramidal neurons increase their size beyond the first month.

Membrane time constant also decreased with postnatal age and showed a temporal course similar to that of input resistance (Figures 2B,G). As for the input resistance, the raw data of the time constant fitted a single exponential decay (r = 0.88; τ = 10.92 days), occurring the 63% of the total decrease in the first two postnatal weeks, as it is shown by the time constant of the fit. Thus, we demonstrated a significant drop in this parameter between newborn and infantile group. Plus, comparisons between the means of age groups demonstrate differences between infantile and young adult group and newborn and young adult group too, without any differences between the two last groups (Figure 2G and Table 1). To know whether time constant decreased as a function of input resistance, we studied the co-variation between both parameters for the whole population. A significant well-fitted linear relationship was found between the two parameters (r = 0.86; *p* < 0.0001: Figure 2H).

### Characteristics of the Action Potentials of Layer V Pyramidal Neurons From the Motor Cortex During Postnatal Development

**FIGURE 3:**
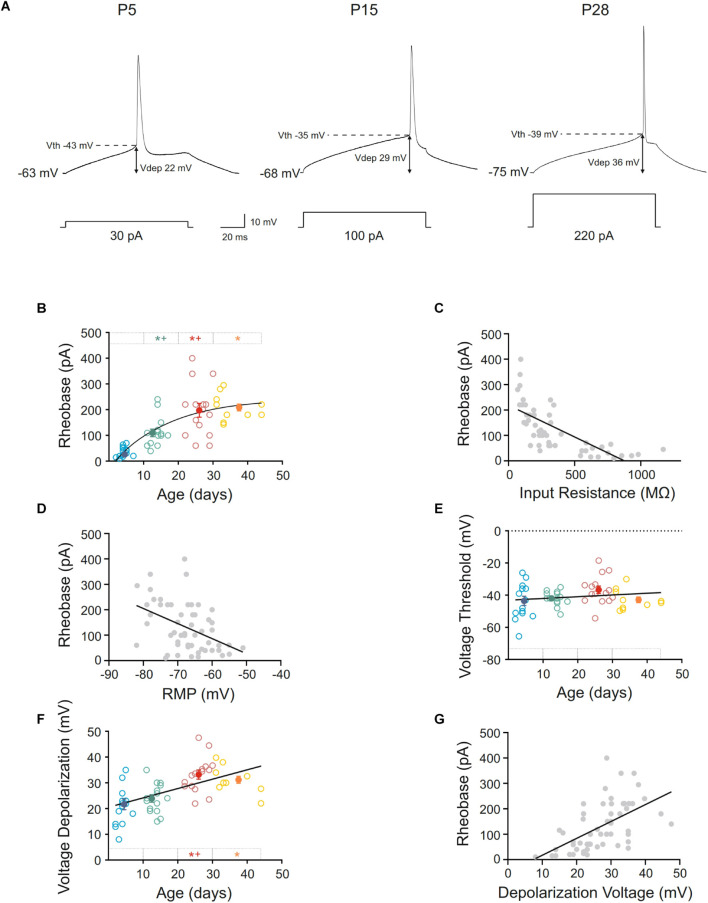
Postnatal development of rheobase and threshold and depolarization voltages of motor cortex pyramidal neurons. **(A)** Membrane voltage responses to the minimum current (rheobase) required to evoke an action potential in representative neurons of different postnatal ages. **(B,E,F)** Scatter plots showing the changes in rheobase **(B)**, voltage threshold **(E),** and voltage depolarization **(F)** as a function of postnatal age. Open symbols represent the raw data, while filled symbols are the means of the four different age groups and their standard errors. Each group is represented by a color (blue = newborn, *n* = 15; green = infantile, *n* = 15; red = young adult, *n* = 15; yellow = adult, *n* = 10). An asterisk indicates a significant difference from the newborn group; a cross indicates a difference between adjacent groups. The significance level was established as *p* ≤ 0.05. **(C,D,G)** Correlation between rheobase and input resistance, r = –0.71; *p* < 0.0001 **(C)**, rheobase and RMP, r = –0.44; *p* < 0.0008 **(D)** and rheobase, and depolarization voltage, r = 0.56; *p* < 0.0001 **(G)**.

The rheobase or minimum current able to elicit an action potential increased during postnatal development, the best fit adjusting a single exponential decay (r = 0.74; τ = 17.33). Indeed, neurons from infantile group displayed a rheobase more than 3-fold higher than the newborn ones. In young adult and adult groups, the rheobase was almost 6-fold higher than in this youngest group. Statistical differences were found in the comparisons between the first three age groups being the last one only statistically different with respect to the control group (Figures 3A,B and Table 1). The increase in rheobase presented a quite similar temporal course, although delayed, respect to the decrease in input resistance, accordingly, we analyzed if these two parameters co-varied during postnatal development. A significant linear relationship was found between these two parameters (r = –0,71; *p* < 0.0001; Figure 3C).

As well as input resistance, other parameters such as membrane potential, depolarization voltage or voltage threshold could be influencing the rheobase. As mentioned above, resting membrane potential was hyperpolarized during development and furthermore, it could be contributing to the rheobase increase. When we analyzed the relationship between these parameters a weak but significant correlation was found (r = −0.44; *p* = 0.0008; Figure 3D), suggesting an influence of the membrane potential to the rheobase decrease during development.

Regarding voltage threshold and depolarization voltage, the first cannot be influencing the change in rheobase since we have described that it is established at birth (Figures 3A,E and Table 1). About the latter, we found that there was an increase in this parameter during development, this increase being statistically significant when newborn (21.67 ± 2.05 mV) or infantile (23.90 ± 1.46 mV) groups were compared with the oldest one (33.2 ± 1.78 mV). No differences were found between the two older groups (Figures 3A,F and Table 1), meaning that depolarization voltage was established at the end of the first month. Figure 3F shows the depolarization voltage values for each neuron along the postnatal days (open symbols) and those raw data fit a linear regression with a slope of 0.37 mV/day (r = 0.54; *p* < 0.0001). According to this, the increase in depolarization voltage could be underlying the increase in rheobase and a good relationship was found when we analyzed the correlation between both parameters during development (r = 0.56; *p* < 0.0001: Figure 3G).

**FIGURE 4:**
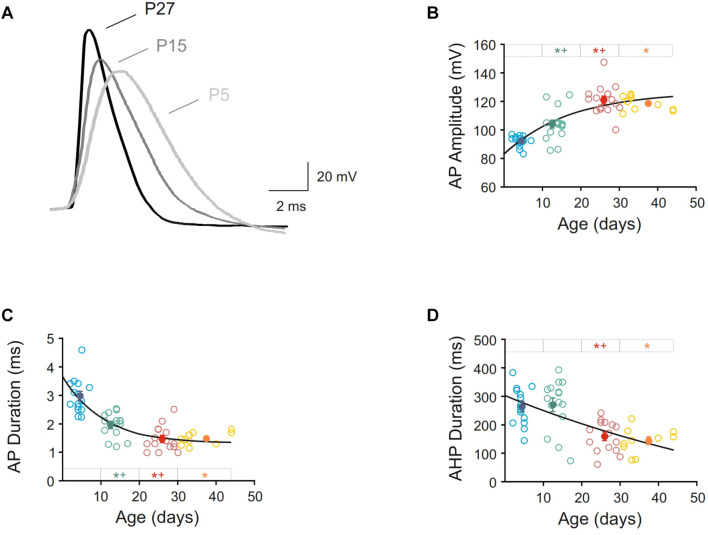
Postnatal development of action potential characteristics of motor cortex pyramidal neurons. **(A)** Membrane voltage responses to suprathreshold stimuli in three representative neurons from newborn, infantile and young adult age groups. Action potentials were synchronized at the beginning of the rising phase to show the differences in amplitude and duration. **(B–D)** Scatter plots showing the amplitude of action potential **(B)**, duration of action potential **(C),** and duration of afterhyperpolarization phase or AHP **(D)** as a function of postnatal age. Open symbols represent the raw data, while filled symbols are the means of the four different age groups and their standard errors. Each group is represented by a color (blue = newborn, *n* = 15; green = infantile, *n* = 15; red = young adult, *n* = 15; yellow = adult, *n* = 10). An asterisk indicates a significant difference from the newborn group; a cross indicates a difference between adjacent groups. The significance level was established as *p* ≤ 0.05.

Furthermore, action potential duration and amplitude and AHP duration were measured, and intense changes were found (Figure 4 and Table 1). Action potential amplitude increased during postnatal maturation, fitting a single exponential decay (r = 0.78; τ = 17.44) and being statistically significant between newborn (92.40 ± 0.96 pA), infantile (104.10 ± 2.92 pA) and young adult (121.10 ± 2.69 pA) groups when compared to each other (Figures 4A,B). As illustrated in Figure 4B and showed by the fitting time constant (τ = 17.44 days), major changes take place during the first three weeks and no changes were observed between the two older groups. Action potential duration decreased during development and significant differences were found between newborn, infantile and young adult groups. Nevertheless, the oldest group did not differ from the young adult one, also maturing this parameter in the first month after birth (Figures 4A,C and Table 1). Finally, the AHP duration also decreased with age being this reduction statistically significant when newborn (264.68 ± 16.60 ms) or infantile (270.44 ± 23.49 ms) groups were compared with the adult one (159.39 ± 13.85 ms). No differences were found neither between newborn and infantile groups nor between young adult and adult groups for this parameter (Figure 4D and Table 1). The raw data along age fit a linear regression with an adjustment of r = 0.60 (slope = −4.42 ms/day; *p* < 0.0001).

### Repetitive Firing Properties of Pyramidal Neurons From the Motor Cortex During Postnatal Development

**FIGURE 5:**
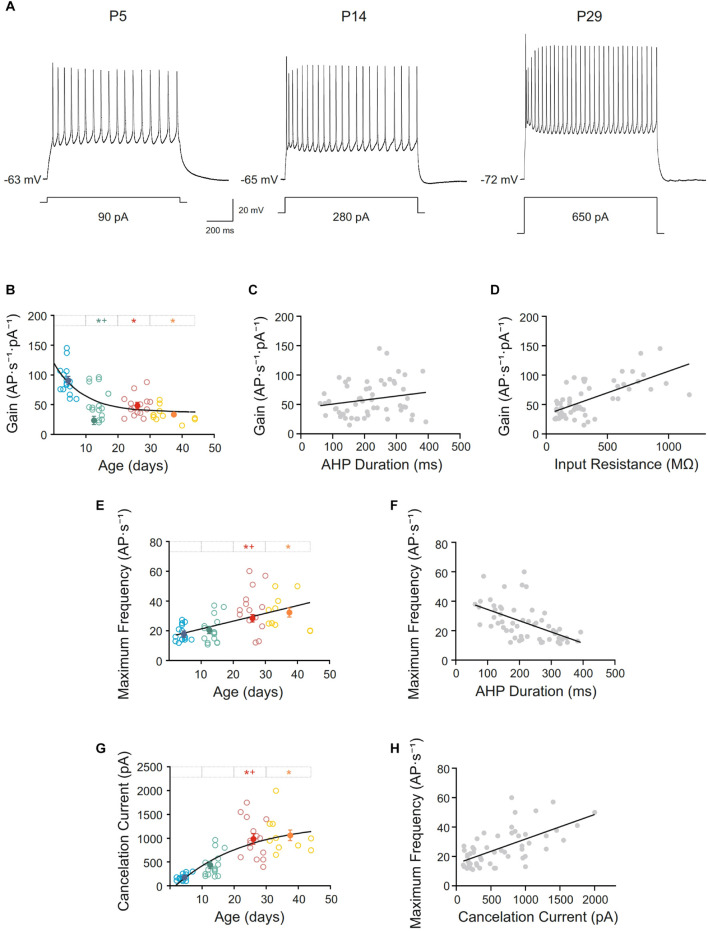
Postnatal development of repetitive firing properties of motor cortex pyramidal neurons. **(A)** Membrane voltage responses to long-lasting current pulse (1s) in representative neurons of different ages. **(B,E,G)** Scatter plots showing the firing frequency gain **(B)**, maximum frequency **(E),** and cancelation current **(G)** as a function of postnatal age. Open symbols represent the raw data, while filled symbols are the means of the four different age groups and their standard errors. Each group is represented by a color (blue = newborn, *n* = 15; green = infantile, *n* = 15; red = young adult, *n* = 15; yellow = adult, *n* = 10). An asterisk indicates a significant difference from the newborn group; a cross indicates a difference between adjacent groups. The significance level was established as *p* ≤ 0.05. **(C,D,F,H)** Correlation between gain and the afterhyperpolarization phase (AHP) duration, r = 0.20, *p* = 0.14 **(C)**, gain and input resistance, r = 0.67, *p* < 0.0001 **(D)** and maximum frequency and AHP duration, r = –0.54, *p* < 0.0001, **(F)** and maximum frequency and cancelation current, r = 0.64, *p* < 0.001 **(H)**.

All recorded pyramidal neurons were silent at resting membrane potential and repetitively discharged when long lasting current pulses were applied. Neurons from this study showed a regular spiking discharge with some grade of adaptation and reached a firing frequency plateau at high intensity currents (Figure 5A). To know if repetitive firing properties were established or not at birth several parameters were studied. Thus, frequency gain was measured and a decline in this parameter was found with age. The raw data for this parameter fitted a single exponential decay (r = 0.71). The fitting time constant shows that major changes occur before the beginning of the second week (τ = 8.81 days; Figure 5B). Statistical analyses were consistent with these findings, demonstrating that the diminution was significant in the second group of age, with any posterior changes (Figures 5A,B and Table 1). Subsequently, the frequency gain matured in the first two postnatal weeks. Frequency gain could be influenced by several parameters, being the AHP duration one of the most important; however, no correlation was found between these membrane properties during development (r = 0.20 and *p* = 0.14; Figure 5C). Otherwise, one possible explanation of the decrease in frequency gain is the demonstrated fall in input resistance, subsequently, we wondered whether their co-varied. A significant well-fitted linear relationship was found between these two parameters (r = 0.67; *p* < 0.0001; Figure 5D).

Additionally, the maximum frequency of the discharge of motor cortex neurons incremented with age. Neurons from adult and young adult groups reached a maximum frequency almost 2-fold higher than neurons from the newborn and infantile groups (Figure 5A). Raw data for this parameter is represented by open symbols in Figure 5E, showing that this parameter increased linearly with age, with a slope of 0.52 AP ⋅ s^–1^/day (r = 0.51; *p* < 0.0001). However, even this linearity, statistical analysis of the age group means (filled symbols in Figure 5E and Table 1) showed differences between infantile and young adult groups, implying that this parameter matured within the second and the third weeks of development. No significant differences were found between newborn and infantile groups or between the last two groups. This increase in maximum frequency presented a temporal course close to the decrease on AHP and a good correlation was found between them (r = −0. 54; *p* < 0.0001; Figure 5F).

Finally, cancelation current was also analyzed, showing an increase during postnatal development from 174.33 ± 17.24 pA in newborn group to 1060.00 ± 101.62 pA in adult group (Filled symbols in Figure 5G and Table 1). The best fit for the raw data was a single exponential decay (r = 0.75; τ = 21.58 days). Regarding group means, this increase was significant between the second and third groups, which is consistent with a fitting time constant of 21.58 days (see Figure 5G), reaching the parameter a stable level from that moment on, without statistical differences between the third and the fourth groups (Figures 5A,G and Table 1). This rise in cancelation current could also be underlying the increase in maximum frequency and, in fact, both parameters showed a good correlation during development (r = 0.64; *p* < 0.001; Figure 5H).

## Discussion

This work shows that the passive membrane properties of layer V pyramidal neurons of the motor cortex are not established at birth. On the contrary, they evolve along the first month after birth. We also show that the characteristics of the action potentials as well as the repetitive firing properties in these neurons are refined during postnatal development leading to a reduction in neuronal excitability and to an increase in the working range in adult neurons, allowing more sensitive and accurate responses.

### Methodological Considerations

Pyramidal neurons of layer V were identified and differentiated from non-pyramidal cells using soma morphology in IR-DIC before the patch recording. In addition, some of the neurons were intracellularly injected and post-patch identified, being all of them pyramidal neurons. Besides, pyramidal neurons were also identified by their firing behavior characterized by an accommodating regular spiking discharge. Non-pyramidal cells were classically described as fast spiking cells that display tonic discharges of fast action potentials with no accommodation. However, other two populations of non-pyramidal cells have been described. One of them is characterized by an irregular spiking with bursts of action potentials at an irregular frequency and the other population displayed a firing behavior characterized by a marked accommodation. This latter population of non-pyramidal cells could be confused with pyramidal neurons. However, they can be differentiated by other electrophysiological parameters as the presence of a rebound of action potentials after a hyperpolarizing current pulse or larger amplitude of AHP (Cauli et al., 1997). Nevertheless, we cannot discard the possibility that a few of these cells could be included as pyramidal neurons in our work.

Furthermore, no bursting discharge was observed in our work for pyramidal neurons so we cannot differentiate by their firing behavior, as in other studies, between cortico-cortical projecting pyramidal neurons (regular spiking neurons) and pyramidal neurons projecting to subcortical regions (burst neurons) (Molnár and Cheung, 2006). This could be due to the fact that in our study the period between the induction of the anesthesia and the decapitation usually did not exceed 2–4 min and, as has been shown, under this condition cells did not display differences in their action potential behavior (Christophe et al., 2005). This short period of anesthesia did not affect the electrophysiological properties of the cells either, since recordings with and without anesthesia were performed in the infantile group with similar results.

### Passive Membrane Properties of Layer V Pyramidal Neurons From the Motor Cortex Are Not Established at Birth

The present results show a hyperpolarization of the resting membrane potential during postnatal development. This fact seems to be a general trend among neocortical pyramidal neurons (Zhu, 2000; Zhang, 2004; Christophe et al., 2005; Etherington and Williams, 2011; Kroon et al., 2019) since in other motor neurons such as genioglossal, oculomotor and spinal it remains constant (Nunez-Abades et al., 1993; Carrascal et al., 2006; Smith and Brownstone, 2020). This drift could be explained by, at least, three different mechanisms. First, a developmental increase of K^+^ leak conductances. Second, a postnatal GABA shift from depolarizing to hyperpolarizing, given by a greater activity of the Na^+^/K^+^/2Cl^–^ cotransporter (NKCC1) in younger neurons and a higher expression of the K^+^/Cl^–^ cotransporter (KCC2) in mature ones (Yamada et al., 2004; Kilb et al., 2013; Luhmann et al., 2014; Peerboom and Wierenga, 2021). Third, it has been proven that tonic GABAergic currents produce membrane hyperpolarization (Torres-Torrelo et al., 2014). Therefore, an increase in GABAergic synaptic input, efficiency and/or in the number of extrasynaptic GABA_*A*_ receptors during development could explain, at least in part, the membrane potential hyperpolarization observed in the neurons of this study.

Furthermore, input resistance decreased during the first postnatal month, especially during the first two weeks, in pyramidal motor neurons. This decline is a characteristic common to most neuronal populations, including not only pyramidal and motor neurons, but also other populations. However, in genioglossal and oculomotor nucleus this parameter is already established at the second postnatal week, while in spinal and neocortical neurons continue to decrease during the whole month (Nunez-Abades et al., 1993; Perez Velazquez and Carlen, 1996; Vincent and Tell, 1997; Ahuja and Wu, 2000; Belleau and Warren, 2000; Zhu, 2000; Murphy and du Lac, 2001; Zhang, 2004; Christophe et al., 2005; Carrascal et al., 2006; Etherington and Williams, 2011; Smith and Brownstone, 2020). The decrease in input resistance could be due to an increase or redistribution of the K^+^ leak channels as well as GABAergic synaptic inputs (Cameron and Nunez-Abades, 2000; Torres-Torrelo et al., 2014). Neuronal size increased during postnatal development as in other studies (Nunez-Abades and Cameron, 1995; Zhu, 2000; Zhang, 2004; Carrascal et al., 2009; Kroon et al., 2019; Smith and Brownstone, 2020) and it could also be the underlying cause of the decrease in input resistance found in our study. In fact, a relationship between input resistance decay and neuronal size increment was found except for genioglossal motoneurons where neurons grow during the 3rd week (Nunez-Abades et al., 1994). In our populations the relationship between input resistance and capacitance, an indirect measure of neuronal size, was weak meaning that the increase in size is not the sole cause of input resistance decrease. The time constant, that reflects integrative properties of the cells, also decreased in pyramidal motor neurons during postnatal maturation, showing a strong correlation with the input resistance decay. This finding is also consistent with those reported for the aforementioned neuronal populations, occurring mainly during the first two postnatal weeks (Nunez-Abades et al., 1993; Perez Velazquez and Carlen, 1996; Vincent and Tell, 1997; Ahuja and Wu, 2000; Belleau and Warren, 2000; Murphy and du Lac, 2001; Zhang, 2004; Carrascal et al., 2006; Etherington and Williams, 2011; Smith and Brownstone, 2020) although in our population the time constant continues to decrease during the whole month, being the parameter stable from this date on. Finally, it has been found that the number of neurons showing voltage rectification is more than 10 fold higher in adults than in neonates, this tendency being common to most studied neurons. The increasing frequency of sag with age found in here could be parallel to a larger density of channels carrying inward rectification currents (Ih). This current is largely carried by sodium ions, and it is activated by a hyperpolarizing input, allowing the repolarization of the cell membrane (Berger et al., 1996; Carrascal et al., 2015).

### The Action Potential Characteristics Are Refined During Postnatal Development

Rheobase increased in motor cortex pyramidal neurons during postnatal development as much as in other neocortical areas (Zhu, 2000; Etherington and Williams, 2011; Kroon et al., 2019). These changes in rheobase implies a decrease in developmental neuronal excitability (Nunez-Abades et al., 1993; Etherington and Williams, 2011). However, different trends in rheobase modifications with postnatal maturation have also been reported in other motor and non-motor neuronal pools. Thus, the rheobase increases in accumbens (Belleau and Warren, 2000), decreases in oculomotor (Carrascal et al., 2006), and is already established at birth in dorsal horn neurons (Baccei and Fitzgerald, 2005). These results lead to the suggestion that the postnatal development of this parameter depends on each neuronal pool. Regarding voltage threshold and voltage depolarization, the present study has shown that, while the former was established at birth, the latter increased with the developmental process. Voltage threshold also remains constant in genioglossal and spinal neurons (Nunez-Abades et al., 1993; Smith and Brownstone, 2020) while it decreases in oculomotor, prefrontal, and visual neurons (Zhang, 2004; Christophe et al., 2005; Carrascal et al., 2006; Etherington and Williams, 2011; Kroon et al., 2019). Voltage depolarization remains constant in some populations such as prefrontal pyramidal neurons and genioglossal motoneurons (Nunez-Abades et al., 1993; Zhang, 2004) and decreases in others such as vestibular neurons (Straka et al., 2005; Carrascal et al., 2006).

The present results show, as in all studied pyramidal neurons from layer V, an increase in the action potential amplitude and a decay in the action potential duration and AHP (Zhu, 2000; Zhang, 2004; Christophe et al., 2005; Etherington and Williams, 2011). The decrease in action potential duration and AHP is also demonstrated in non-pyramidal cells, being firstly established in genioglossal motoneurons (Nunez-Abades et al., 1993; Vincent and Tell, 1997; Murphy and du Lac, 2001; Carrascal et al., 2006; Smith and Brownstone, 2020). Nevertheless, the increase in the amplitude of action potential during development found in here and other studies (Etherington and Williams, 2011; Kroon et al., 2019) is not universal among neuronal pools because in other populations this parameter is already established at birth such is genioglossal and oculomotor motoneurons (Nunez-Abades et al., 1993; Carrascal et al., 2006). The refinement in action potential shape is mainly given by an increase in density and/or a faster activation of the voltage-gated Na^+^ and K^+^ channels (Nunez-Abades et al., 1993; Carrascal et al., 2006, 2015; Smith and Brownstone, 2020). A decrement in voltage-activated Ca^2+^ channels could also contribute to the decrease in spike duration, and these conductances could also be responsible of the decay in AHP duration, since this phase is dependent on Ca^2+^-dependent K^+^ currents (Nunez-Abades et al., 1993; Carrascal et al., 2006).

### Repetitive Firing Properties of Pyramidal Neurons From the Motor Cortex Are Adjusted During Postnatal Development

Pyramidal neurons from the primary motor cortex underwent wide modifications in their repetitive firing properties, a measurement of neuronal responsiveness. It can be stated that all the neuronal populations studied to date modified their firing properties, although with different patterns of change. Frequency gain decreases in neocortical pyramidal neurons (Zhang, 2004), including those from the present work, as well as in lumbar and cervical motoneurons (Smith and Brownstone, 2020) while increases in other motor (Nunez-Abades et al., 1993; Carrascal et al., 2006) and non-motor pools (Tennigkeit et al., 1998). However, in all studied neurons the maximum frequency of discharge increased with postnatal development (Nunez-Abades et al., 1993; Zhang, 2004; Carrascal et al., 2006; Smith and Brownstone, 2020), although it does not mature at the same time. Thus, while in pyramidal neurons of the motor cortex the maximum firing frequency increases from the third week, in prefrontal pyramidal neurons and genioglossal motoneurons it occurs earlier (Zhang, 2004). This higher firing frequency in adult neurons could be due to the decrease in the duration of action potential and AHP (Smith and Brownstone, 2020). In fact, the good correlation between AHP duration and the maximum firing frequency seen in here indicates that both parameters mature in parallel. Other studies (Nunez-Abades et al., 1993; Carrascal et al., 2006) propose that this increase in maximum firing frequency would be more linked to a faster activation of the delay rectifier or to a shorter inactivation stage of the voltage-gated Na^+^ channels. Regardless of the ionic nature that underlies these changes, this increase in the maximum firing frequency would be influenced by a greater cancelation current in the adult population, which increases the working range of these motor cortex neurons. The combination of increased working range and decreased frequency gain allow neurons to respond with greater precision to a greater range of current intensities (Smith and Brownstone, 2020).

Comparing our study with those reported for other pyramidal neurons, and mentioned above, it seems that the electrophysiological parameters for these neurons maturated during postnatal development in the same way independently of the cortex region and reaching similar final values. Overall, all these changes led to a decrease in excitability in most of the pyramidal and motor neuron populations except in oculomotor nucleus, this increase being consistent with the task of this motoneurons that must reach higher discharge to control eye movement (Carrascal et al., 2010). On the other hand, in genioglossal motoneurons the postanal refinement of electrophysiological properties is established earlier than in the other populations being the spinal and above all the motor cortex populations the last ones. The fact that electrophysiological properties of genioglossal motoneurons were established earlier than those of other populations is also consistent with the task that they develop. This pool of neurons innervates the tongue, a muscle that is implicate in several motor tasks, such as suckling, swallowing, and breathing, tasks that must be operative soon after birth (Nunez-Abades et al., 1993). In contrast, the locomotor function develops later in time, since neither the musculoskeletal system nor the nervous system that innervates it is sufficiently developed in rodents until at least the second-third postnatal weeks, when quadruped ambulation begins (Smith and Brownstone, 2020). Therefore, the maturation of the electrophysiological properties of motor neurons is more related to the specific motor task that they perform than to their location along the rostro-caudal axis.

## Conclusion

The electrophysiological properties of layer V pyramidal neurons from the primary motor cortex are tuned during the first postnatal month, although the time course of these changes depends on each specific electrophysiological parameter. These electrophysiological modifications lead to a decrease in excitability and an increase in the working range. This allows mature neurons to generate more precise and graded responses and contributes to the development of the great variety of specialized neuronal circuits founding the complex behaviors of adult mammals. By comparing the evolution of the different neuronal properties in different motor neuron populations during postnatal development, we hypothesize that the temporal course of the acquisition of motor neuron functional properties does not depend on the anteroposterior localization of the nuclei but on the specific motor task that they perform.

## Data Availability Statement

The raw data supporting the conclusions of this article will be made available by the authors, without undue reservation.

## Ethics Statement

The animal study was reviewed and approved by the Animal Ethics Committee of the University of Seville.

## Author Contributions

PP-G and RP-D: data acquisition and analysis of electrophysiological experiments, discussion of results, and manuscript preparation and figure design. NG-D, RG-O, and SD-G: data analysis, discussion of results, and figure design. CC and PN-A: experimental design, data analysis, discussion of results, and manuscript preparation and writing. LC: conception of the work, experimental design, data acquisition and analysis, discussion of results, manuscript preparation, and writing. All authors contributed to the article and approved the submitted version.

## Conflict of Interest

The authors declare that the research was conducted in the absence of any commercial or financial relationships that could be construed as a potential conflict of interest.

## Publisher’s Note

All claims expressed in this article are solely those of the authors and do not necessarily represent those of their affiliated organizations, or those of the publisher, the editors and the reviewers. Any product that may be evaluated in this article, or claim that may be made by its manufacturer, is not guaranteed or endorsed by the publisher.
